# ABCG5/ABCG8-independent mechanisms fail to maintain sterol balance in mice fed a high-cholesterol diet

**DOI:** 10.1016/j.jlr.2025.100902

**Published:** 2025-09-16

**Authors:** Garrett B. Anspach, Rupinder Kaur, Isha Chauhan, Erika L. Savage, Brittney Poole, Victoria P. Noffsinger, Xiaoming Fu, Zeneng Wang, Clairity Voy, Ryan E. Temel, Scott R. Gordon, Robert N. Helsley, Gregory A. Graf

**Affiliations:** 1 Saha Cardiovascular Research Center, College of Medicine, University of Kentucky, Lexington, KY, USA; 2 Department of Physiology, College of Medicine, University of Kentucky, Lexington, KY, USA; 3 Department of Cardiovascular and Metabolic Sciences, Cleveland Clinic, Cleveland, OH, USA; 4 Department of Internal Medicine, College of Medicine, University of Kentucky, Lexington, KY, USA; 5 Barnstable Brown Diabetes Center, UK Healthcare, Lexington, KY, USA

**Keywords:** ABCG5, ABCG8, cholesterol, xenosterol, phytosterol, bile, transintestinal, TICE, liver, intestine

## Abstract

The ABCG5/ABCG8 (G5G8) sterol transporter opposes the accumulation of dietary xenosterols but is also the primary mediator of biliary cholesterol secretion. In humans and in mouse models of disrupted biliary cholesterol secretion, fecal neutral sterols (FNSs) remain constant, indicating the presence of an alternate pathway for cholesterol excretion. Transintestinal cholesterol elimination or excretion (TICE) is thought to compensate for biliary disruptions and G5G8 insufficiency. We sought to measure the compensatory increase in intestinal cholesterol secretion and provide mechanistic insight for how TICE maintains sterol balance in the absence of hepatic G5G8. Differences were not observed in FNSs between control, acute, and chronic liver-specific G5G8-deficient mice (G5G8^LKO^). Cholesterol content did not differ at any point along the intestinal tract between genotypes. We also observed no change in the expression of apical or basolateral sterol transporters in the proximal small intestine. We then measured biliary and intestinal cholesterol secretion rates using cholesterol-free and cholesterol-enriched bile acid micelles as acceptors. While biliary cholesterol secretion was reduced, the intrinsic rate of intestinal cholesterol secretion did not differ between genotypes. G5G8^LKO^ and whole-body G5G8-deficient mice were challenged with a cholesterol-containing diet. While control mice upregulate FNS excretion, G5G8-independent mechanisms fail to maintain fecal sterol excretion and oppose the accumulation of cholesterol in liver and plasma. These studies indicate that while G5G8-independent mechanisms can mediate cholesterol excretion, TICE is not upregulated in response to a loss of hepatic G5G8 and is unable to compensate for hepatic or whole-body G5G8 deficiency in response to dietary cholesterol in mice.

ABCG5/ABCG8 (G5G8) is an obligate heterodimer expressed in the liver and intestine that promotes the secretion of sterols into bile and the intestinal lumen, respectively (1, 2, 3, 4, 5, 6). Mutations in either ABC half-transporter cause sitosterolemia (*STSL*, OMIM: #210250 and #618666), a rare recessive disorder characterized by the accumulation of noncholesterol sterols (xenosterols) in plasma and tissues (7, 8). The clinical presentation of the disease is varied and includes hemolytic disorders and thrombocytopenia but not necessarily hypercholesterolemia (reviewed in Ref. (9)). However, multiple genome-wide association studies have linked rare and common variants in *ABCG5* and *ABCG8* to elevated plasma levels of total and LDL cholesterol and coronary artery disease, suggesting that while *STSL* is a recessive disorder, loss-of-function variant or *ABCG5 ABCG8* haploinsufficiency contributes to elevated plasma cholesterol and coronary artery disease risk (10).

An estimated 70% of the body’s cholesterol is synthesized in the liver and delivered to peripheral cells in apolipoprotein B-containing lipoproteins. Nonsteroidogenic peripheral cells lack the ability to metabolize excess cholesterol, thereby necessitating a pathway for its return to the liver for metabolism to primary bile acids or direct secretion into bile and elimination from the body (11). G5G8 is the principal mediator of biliary cholesterol secretion in the final step of the reverse cholesterol transport (RCT) pathway and accounts for 70–80% of biliary cholesterol secretion (2, 5, 6). However, genetic inactivation of *Abcg8* in mice does not result in a reduction in fecal sterol excretion (12). Similarly, disruptions in biliary cholesterol secretion via inactivation of bile acid secretion or bile duct ligation do not reduce, and, in some cases, increase fecal neutral sterols (FNSs). This alternate pathway for elimination of excess cholesterol has been termed transintestinal cholesterol elimination or excretion (TICE) (13).

Stable isotope studies suggest that TICE accounts for approximately 30% of whole-body cholesterol excretion in mice maintained on standard rodent diets (14). However, TICE can be increased by a number of pharmacological stimuli, including statins, fibrates, and agonists of the liver-X and farnesoid X receptors (14, 15, 16, 17, 18). Under these conditions, TICE can account for greater than 60% of cholesterol excretion in mice. Evidence also supports a role for TICE in humans, where it is estimated to account for 35% of cholesterol excretion (16, 19). However, the mediators of intestinal cholesterol uptake from the plasma compartment and G5G8-independent transport across the apical surface of intestinal enterocytes have remained elusive. Studies of LDL, HDL, and their receptors on the basolateral surface have produced mixed results (13, 16, 20, 21, 22, 23, 24). While the complete absence of G5G8 has no effect on sterol balance, it was shown to be essential for pharmacologically stimulated TICE (14, 25). While the role of G5G8 in opposing dietary xenosterol accumulation is clear, the dispensability of G5G8 in cholesterol metabolism is less certain.

Either hepatic or intestinal G5G8 is sufficient to prevent the accumulation of dietary xenosterols, but the tissue-specific role of G5G8 in the maintenance of cholesterol balance has not been investigated (26). In the present study, we characterized the adaptive response in the intestine to hepatic G5G8 deficiency that maintains FNS excretion. Hepatic G5G8 was inactivated in mice using a germline liver-specific Cre recombinase transgene and acutely using an adeno-associated viral vector (AAV). Following acute hepatic G5G8 deletion, the maintenance of FNS excretion was immediate. Intestinal cholesterol content was not reduced in the proximal small intestine, despite the reduction in biliary cholesterol secretion. In perfusion studies, the intrinsic rate of intestinal cholesterol secretion did not differ between genotypes. Finally, we challenged whole-body and liver-specific G5G8-deficient mice with a cholesterol-containing diet. TICE failed to maintain fecal excretion of cholesterol with compromised biliary cholesterol secretion or whole-body G5G8 deficiency. These studies indicate that the loss of hepatic G5G8 does not result in the upregulation of TICE to maintain cholesterol excretion in mice maintained on standard rodent diets and that TICE is incapable of maintaining fecal cholesterol excretion in mice challenged with a high-cholesterol diet.

## Materials and methods

### Animal care and use

All animal procedures were approved by and performed under the supervision of the University of Kentucky Institutional Animal Care and Use Committee. The colony is maintained in a specific pathogen-free, temperature-controlled (21°C) facility with a 14/10 light/dark cycle. Mice were group housed in individually ventilated cages with P.J. Murphy Coarse SaniChip bedding with free access to a cereal-based diet (2918 Teklad Irradiated Rodent Diet [18% protein, 6% fat, 44% carbohydrate kcal/wt]) and water unless otherwise indicated. Experimental mice were generated using a trio mating scheme, weaned between 18 and 21 days of age, and enrolled in experiments between 8 and 12 weeks of age. Male and female littermates were included in all experiments unless otherwise indicated. All mice were monitored for appearance of coat condition, weight, and mobility on a weekly basis. Experimental mice were randomized to treatment groups, and groups were deidentified during biochemical analyses. Conventional *Abcg5 Abcg8*-deficient mice (strain #004670) and mice harboring lox-p sites flanking exon 1 of *Abcg5* and exons 1 and 2 of *Abcg8* (*Abcg5 Abcg8*^fl/fl^ strain #026702) are maintained in our breeding colony and undergo routine strain refreshment against the C57Bl6/J strain (strain #000664; The Jackson Laboratory, Bar Harbor, ME) every five to six generations.

### Acute and chronic G5G8 deficiency

*Abcg5 Abcg8*^fl/fl^ female mice were crossed with the C57Bl6/J males harboring a Cre recombinase transgene driven by the mouse albumin promoter (strain #003574) to generate control and G5G8 liver-specific G5G8 knockout mice (G5G8^LKO^). To accomplish acute inactivation of G5G8 (G5G8^LKO-A^), *Abcg5 Abcg8*^fl/fl^ mice were administered a control AAV (serotype 2/8) or an AAV8 encoding Cre recombinase under the control of the thyroxine-binding globulin promoter (AAV8_TBG-Cre; Penn Vector Core) at doses of 5 × 10^11^ gc/25 g mouse via tail vein. At 8 weeks of age, mice were single housed in wire-bottom cages and feces were collected 3 days prior to and on days 3, 5, 7, 14, and 28 following AAV delivery. On day 28, mice were anesthetized under 3% isoflurane (Covetrus; 11695-6777-2), the common bile duct was ligated, and the gallbladder was cannulated and diverted to a collection tube for 30 min (basal bile). Mice were euthanized via cardiac puncture and liver, and five equal segments (∼6 cm) of small intestine were excised, flash frozen, and stored at −80°C until analysis. An independent cohort of control and G5G8^LKO^ mice was euthanized at 12 weeks of age in the postprandial phase (“lights-off” + 6 h), and five equal segments of the small intestine, cecum, and colon and their contents were dissected and immediately extracted for lipid analysis.

Independent cohorts of mice comprised of control and G5G8^LKO^ and WT and conventional *Abcg5 Abcg8*-deficient (G5G8^−/−^) mice were sequentially fed purified diets in the absence and presence of excess cholesterol. Control and G5G8^LKO^ mice are maintained on a standard rodent diet. At 8 weeks of age, the mice were fed a custom plant sterol-free (PS-free) diet (#D10040301; 21 kcal% protein, 61 kcal% carbohydrate, 18 kcal% fat [lard]) for a period of 2 weeks. Mice were then fed this same diet supplemented with 0.2% cholesterol (w/w). Feces were collected for the final 3 days on each diet. To avoid confounding effects of bioactive phytosterols that accumulate in mice lacking *Abcg5 Abcg8*, this strain is maintained on the PS-free diet. Baseline feces were collected, and the mice were placed on the cholesterol-supplemented PS-free diet for 2 weeks. The mice were then placed on a standard diet for 2 weeks. As with the floxed strain, feces were collected for the final 3 days on each diet. At termination, basal bile was collected and tissues dissected.

### Simultaneous determination of biliary and intestinal cholesterol secretion

Secretion of cholesterol from the liver and intestine was determined as described previously (15, 24). Briefly, mice were anesthetized, the common bile duct ligated, and bile diverted to collection tubes. Basal bile was collected for 30 min to deplete the endogenous bile acid pool. During that collection period, the tail vein was fitted with a tail vein catheter connected to a syringe pump. The proximal 10 cm of the small intestine was fitted with inflow and outflow catheters, flushed with Krebs Henseleit Buffer, and connected to a peristaltic pump. Following basal bile collection, taurocholate was infused (100 nmol/min), and the proximal small intestine was perfused with Krebs Henseleit Buffer containing bile acid micelles (10 mM taurocholate, 2 mM phosphatidylcholine, and ±0.32 mM cholesterol) at a rate of 14 μl/min.

### Analysis of mRNA and protein

The relative abundance of transcripts was determined by real-time PCR as previously reported (Table S1) (15, 27). RNA was isolated from ∼100 mg of tissue using RNA Stat-60 phenol chloroform (TEL TEST CS502; Fisher Scientific; NC9489785) and purified using the Qiagen RNeasy Mini Kit (Qiagen; 74106). RNA (250 ng) was converted to complementary DNA (cDNA) using the iScript cDNA synthesis kit (Bio-Rad; 170-8891), cDNA was diluted 1:40, and RT-PCR was performed with primer pairs directed toward the indicated transcript (supplemental Table S1) and the SYBR Green detection system (Life Technologies; 4364346). Data were normalized to threshold cycle values for GAPDH and hypoxanthine-guanine phosphoribosyltransferase and the mean of the indicated control group and expressed as fold change by the ΔΔCt method. The relative abundance of Abcg5 and Abcg8 protein was determined by immunoblot analysis using validated antibodies developed in-house (28, 29).

### Analysis of hepatic, plasma, and biliary lipids

Hepatic lipids were extracted, resuspended in 1% Triton X-100, and cholesterol content determined using commercial assay as previously described (27). Total plasma and biliary cholesterol were determined using a commercially available enzymatic colorimetric assay (FujiFilm Wako). Bile was diluted 10-fold, and bile acids were measured using a previously published stable isotope dilution LC–MS/MS methodology with slight modification (30). A C18 column (Kinetex® 2.6 μm Polar, 50 × 2.1 mm; Phenomenex) was used to separate bile acids, with a binary gradient consisting of 0.1% propionic acid in water and 0.1% acetic acid in methanol.

### Fecal and intestinal cholesterol

Promptly after excision from the mouse, the whole tissue (including lumenal content) was collected in preweighed glass tubes primed with an internal standard of 5-α cholestane. Tubes were reweighed with tissue, and Folch reagent (2:1 chloroform:methanol, 2 ml) and KOH (50%, 200 μl) were added. The solution was incubated at 37°C overnight and vortexed periodically. Hexanes (2 ml) were added to the tube and vortexed, followed by the addition of water (2 ml), vortexed, and centrifuged for 10 min at 10,000 rpm to separate aqueous and organic phases. The top organic phase was transferred to a clean vial, and the aqueous phase was re-extracted with hexanes (2 ml). An aliquot of total tissue extract (100 μl) was dried down, and *N*-methyl-*N*-(trimethylsilyl)trifluoroacetamide (30 μ) was added and heated at 95°C for 1 h. Ethyl acetate (70 μl) was added and analyzed by injecting 5 μl of sample onto an HP-5MS (0.250-mm inner diameter × 30 m × 0.25 μm) ultra-inert gas chromatography column (Agilent; 19091S-433UI) at 250°C and installed in an Agilent Technologies 7890B gas chromatograph equipped with an Agilent Technologies 7693A autosampler using on-column injection and a Mass Selective Detector (Agilent G7081B). Cholesterol was quantified using the area under the curve normalized to 5α-cholestane.

Feces were dried, weighed, and ground to a fine powder. Approximately 100 mg of feces was added to glass tubes primed with 25 μg 5-alpha-cholestane, and FNS were extracted in 2 ml of 2:1 chloroform:methanol, and 200 μl of 50% KOH was added. The solution was incubated at 37°C overnight and vortexed periodically. Two milliliters of hexanes were added to the tube and vortexed. Two milliliters of water were added, the sample was vortexed, and centrifuged for 10 min at 10,000 rpm for phase separation. The top organic phase was transferred to GC vials, dried, resuspended, and analyzed by GC-MS.

### Human intestinal samples

Deidentified small intestinal tissue was obtained from the UK Center for Clinical and Translational Science Biospecimen Core. Samples of small bowel were obtained from patients undergoing scheduled surgeries at the University of Kentucky Chandler Medical Center. All patients gave written informed consent, the study was approved by the University of Kentucky IRB (#44026), and the study abides by the Declaration of Helsinki principles. Tissues deemed “healthy” at the margins of the resected bowel segment were trimmed, snap frozen, and banked at −80°C until the time of analysis. The location of the intestinal segment and patient characteristics were obtained from patient records by a research coordinator in the core facility (Table 2). RNA was isolated, reverse transcribed, and analyzed for gene expression by RT-PCR using TaqMan™ Probes according to the manufacturer’s instructions (Applied Biosystems, Waltham, MA).

**Table 2:** Patient characteristics of human intestinal donors

Measure	Duodenum	Jejunum	Ileum
n	11	5	32
Female (%)	27.3	40.0	56.3
BMI	28.2	28.9	29.7
Age	62.2	62.4	62.2
Statin (%)	9.1	20.0	56.3

### Data analysis

Unless otherwise indicated, data are mean ± SEM. For most measures, individual values are shown in addition to summary statistics. Data were analyzed by two-way ANOVA using genotype and sex as factors with Sidak’s post hoc tests to determine the effect of genotype within sex or intestinal segment as indicated. All analyses included tests for normality. Where main effects of sex or a sex-by-genotype interaction were detected in the model, data were analyzed irrespective of sex using a Student’s *t*-test. Males are denoted by filled symbols, and females are denoted by open symbols. For intestinal and cholesterol secretion rates, cumulative cholesterol secretion was analyzed by simple linear regression within each sex and the effect of genotype determined by difference in slope.

## Results

**Fig. 1:**
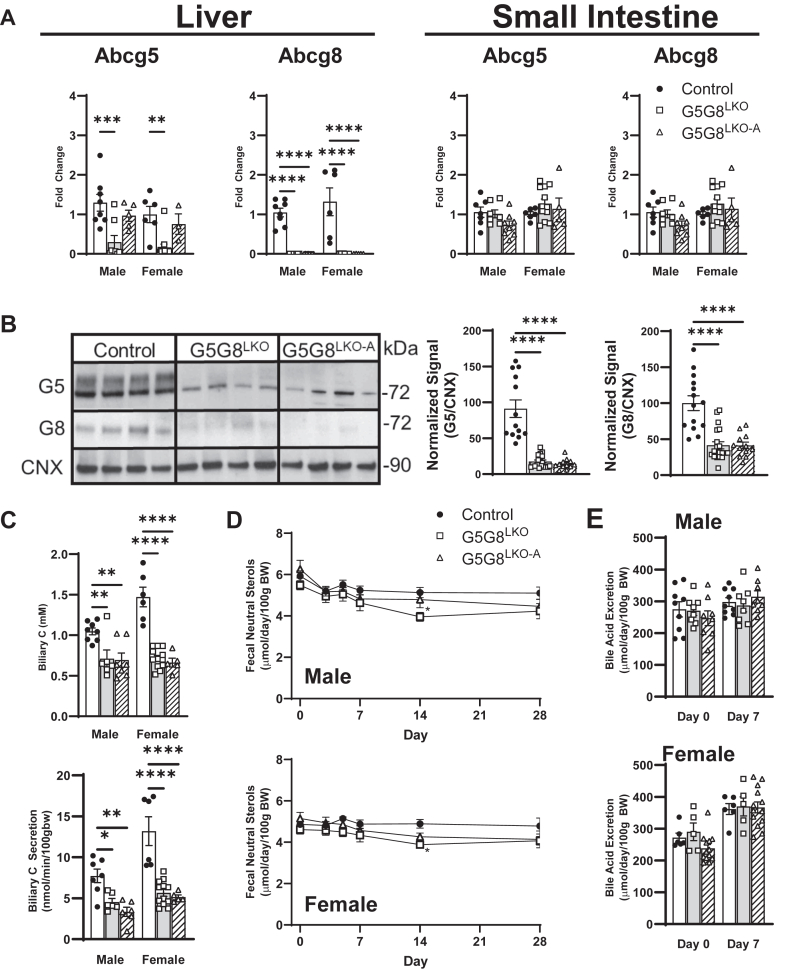
The impact of chronic or acute deletion of Abcg5/Abcg8 on biliary cholesterol secretion and fecal sterol excretion. Mice harboring floxed *Abcg5 Abcg8* were bred to the Alb-Cre strain (G5G8^LKO^) and maintained on a standard diet to 8–10 weeks of age. Alb-Cre-negative mice were delivered control AAV (control) or AAV_TBG-Cre (G5G8^LKO-A^) to accomplish acute inactivation of hepatic G5G8. All mice were euthanized 28 days following AAV delivery. A: Relative abundance of Abcg5 and Abcg8 mRNA in liver and proximal small intestine. B: Detection of Abcg5 and Abcg8 proteins in liver by immunoblot analysis. C: Biliary cholesterol concentrations in gallbladder bile and biliary cholesterol secretion rate in basal bile at termination of the experiment. D and E: Fecal neutral and acidic sterols prior to (day 0) and up to 28 days following AAV delivery. Data are mean ± SEM (n = 6–12) in males and females and analyzed by two-way ANOVA using sex and genotype as factors. Effects of genotype within sex were determined using a Sidak’s multiple comparison test. Sequential measures of fecal neutral sterols (D) were conducted within sex using a two-way repeated-measures ANOVA using genotype and time as factors. A Dunnett’s post hoc test was used to determine differences from day 0. ∗*P* < 0.05, ∗∗*P* < 0.01, ∗∗∗*P* < 0.001, and ∗∗∗∗*P* < 0.0001.

Multiple animal models of impaired biliary cholesterol secretion maintain FNS output, supporting a role for enhanced TICE as an adaptive response. We compared FNS excretion between control, chronic, and acute inactivation of hepatic G5G8 to determine the rapidity of this adaptive response. Hepatic G5G8 was deleted in *Abcg5 Abcg8*^fl/fl^ mice using an albumin-Cre transgene or acutely using AAV8_TBG-Cre while the mice were maintained on a standard rodent diet. Hepatic, but not intestinal, mRNAs for G5 and G8 were reduced in both models compared with control mice (Fig. 1A). Similarly, hepatic G5 and G8 proteins were reduced in both models (Fig. 1B). Differences due to hepatic genotype were not observed for physiological measures, such as body weight, liver weight, and basal bile flow (Table 1). As expected, biliary cholesterol concentrations and cholesterol secretion rates in basal bile were substantially reduced in mice lacking hepatic G5G8 in both males and females (Fig. 1C).

**Table 1:** Physiological measures in control and liver-specific G5G8-deficient mice

Measure	Male			Female		
Group (n)	Control (9)	G5G8^LKO^ (8)	G5G8^LKO-A^ (8)	Control (6)	G5G8^LKO^ (13)	G5G8^LKO-A^ (5)
Body weight (BW; g)^a^	25.61 ± 0.85	24.58 ± 0.53	25.46 ± 0.59	20.15 ± 0.34	19.88 ± 0.37	21.08 ± 0.39
Liver weight/BW (%)^a^	4.44 ± 0.18	4.80 ± 0.20	4.29 ± 0.18	4.81 ± 0.12	5.07 ± 0.12	5.13 ± 0.14
Basal bile flow (μl/min/100 g BW)^a^	6.09 ± 1.12	7.07 ± 0.12	4.80 ± 0.98	8.83 ± 0.67	8.30 ± 0.96	7.88 ± 0.36
Plasma cholesterol (mM)^a^	3.2 ± 0.84	3.28 ± 0.73	2.51 ± 0.37^b^	2.71 ± 0.36	2.35 ± 035	2.31 ± 0.21

a Main effect of sex.

b *P* < 0.05 compared with control.

Baseline FNSs did not differ between groups (Fig. 1D). Nor did they differ for up to 28 days between controls and mice with either chronic or acute inactivation of G5G8. The apparent downward trend in FNS over the 28-day period, particularly in males, is largely due to weight gain during this period as the data are normalized to body weight. We did observe a modest, but statistically significant, reduction in the G5G8^LKO^ strain at the 14-d time point. However, this group lacks G5G8 during embryonic development when the albumin promoter driving expression of Cre recombinase becomes active, and we did not observe differences at any other time point over the course of the experiment. We also did not observe differences in fecal bile acids between groups at the 28-d timepoint (Fig. 1E). These data indicate that TICE can adapt to the loss in hepatic G5G8 within a timescale not discernible under these experimental conditions. In addition, neither a transient reduction nor recovery in FNS in G5G8^LKO-A^ mice was observed. Therefore, we limited our subsequent analysis to control and G5G8^LKO^ mice.

**Fig. 2:**
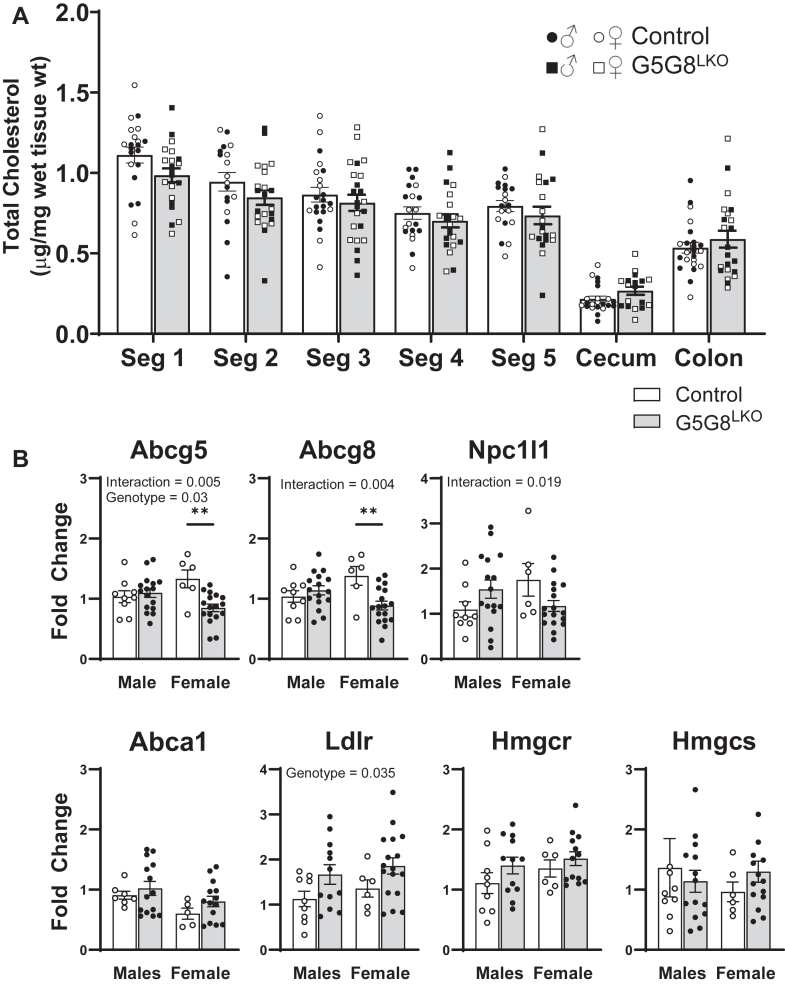
Tissue and lumenal cholesterol content along the gastrointestinal tract in control and G5G8^LKO^ mice. A: Mice were maintained on a standard diet for 12 weeks of age and euthanized in the postprandial phase 6 hours following “lights-off.” The small intestine (five equidistant segments), cecum, and colon were dissected and weighed. Sterols were immediately extracted from whole tissue and lumenal contents and quantified by GC-MS. Areas under the curve for cholesterol were normalized to the internal standard, and cholesterol content was expressed as microgram/milligram wet tissue weight. Data are mean ± SEM in both males (filled symbols) and females (open symbols) and were analyzed by two-way ANOVA using segment and genotype as factors with Sidak’s multiple comparisons tests for the effect of genotype within each segment (n = 20–23). B: The relative abundance of mRNAs encoding sterol-transporting enzymes in the proximal small intestine as determined by RT-PCR. Data are mean ± SEM and were analyzed by two-way ANOVA using genotype and sex as factors (n = 8–12). Bars terminating in horizontal lines indicate the main effect of sex irrespective of genotype. ∗*P* < 0.05.

Next, we reasoned that if biliary cholesterol is disrupted and less cholesterol arrives in the small intestine via the common bile duct, TICE must close this cholesterol excretion gap somewhere along the length of the intestinal tract. Food consumption and biliary lipid output are entrained to the light/dark-fasting/feeding cycle. Ileal fibroblast growth factor 15 levels are greatest 6 h after lights off during the postprandial phase, indicating active biliary cholesterol secretion and delivery of hepatic bile to the small intestine at this time (31). We euthanized a cohort of mice at this time to assess cholesterol content along the length of the gastrointestinal tract across genotypes. The intestinal segments and their contents were excised, weighed, and immediately placed in Folch reagent for lipid extraction. While cholesterol content declined along the length of the intestinal tract, it did not differ in any segment between genotypes (Fig. 2A). These data indicate that the “sterol gap” within the small intestine due to reduced biliary contribution is closed in the proximal segment of the small intestine.

We next evaluated the mRNA expression of sterol-transporting proteins in the first segment of the small intestine in both male and female mice (Fig. 2B). While differences were observed between sexes, the abundance of intestinal Abcg5 and Abcg8 mRNA was unaffected by the loss of G5G8 in the liver. Similarly, mRNA expression of the apical sterol uptake transporter, Npc1l1, and basolateral efflux transporter, Abca1, was also unaltered. While there is an apparent trend toward increased expression of genes regulated by Srebp2 (Ldlr and Hmgcr) consistent with a reduction in cholesterol in the proximal small intestine, none of these differences reached statistical significance within sex. In the absence of a main effect of sex or interaction, these data were also analyzed by a Student’s *t*-test independent of sex. While a statistical difference was detected for Ldlr (*P* = 0.024), this was not the case for other Srepb2 target genes.

**Fig. 3:**
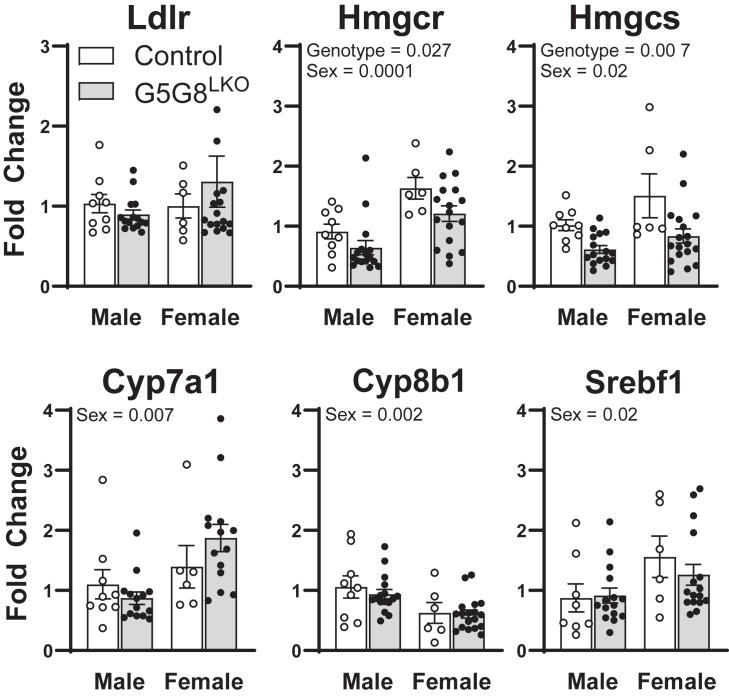
Expression of selected enzymes in lipid metabolism and synthesis in the liver of control and G5G8^LKO^ mice. Total RNA was isolated from the liver of control and G5G8^LKO^ mice (Fig. 1), and the relative abundance of mRNA of selected enzymes was quantified by RT-PCR. Data are mean ± SEM and analyzed by two-way ANOVA using genotype and sex as factors, as with Sidak’s multiple comparisons tests for the effect of genotype within each sex (n = 8–12). Bars terminating in horizontal lines indicate the main effect of sex irrespective of genotype. *P* values for the effect of genotype irrespective of sex are indicated by text. ∗*P* < 0.05, ∗∗*P* < 0.01, ∗∗∗*P* < 0.001, and ∗∗∗∗*P* < 0.0001.

The loss of hepatic G5G8 is expected to promote the accumulation of cholesterol in the liver, reduce synthesis, and increase its metabolism to primary bile acids. The expression of both Hmgcr and Hmgcs was elevated in female mice relative to males and was reduced in G5G8^LKO^ mice irrespective of sex (Fig. 3). However, mRNA for Ldlr was unaffected. We previously published that the loss of G5G8 promoted de novo lipogenesis in mice fed a high-fat diet (32). Srebf1 and its target genes, Fasn and Acc1, were not affected by the loss of hepatic G5G8 in mice maintained on a standard diet. The expression of the rate-limiting enzymes in bile acid synthesis, Cyp7a1 and Cpy8b1, differed by sex but was not affected by the absence of G5G8.

**Fig. 4:**
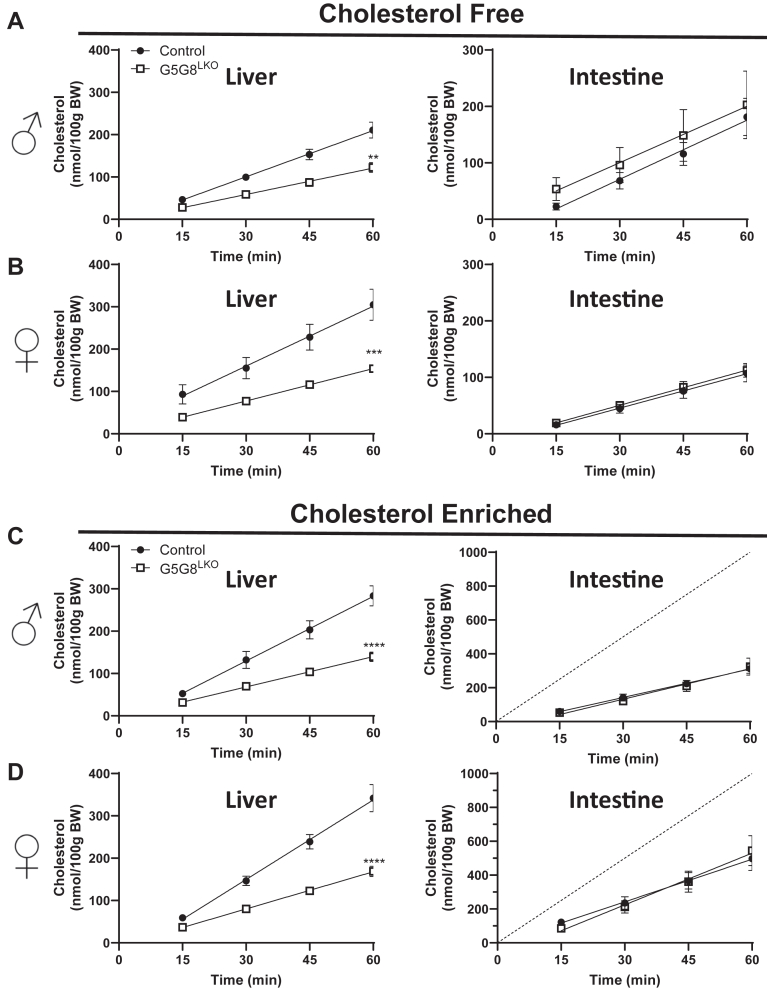
Biliary and intestinal cholesterol secretion in control and G5G8^LKO^ mice. Mice were bred and housed as in Figure 1. The common bile duct was ligated, the gallbladder was cannulated, and bile diverted into collection tubes in both male (A, C) and female (B, D) mice. Bile flow was maintained by continuous tail-vein infusion of TC (100 nmol/min). The proximal small intestine (10 cm) was perfused with Krebs-Henseleit buffer containing mixed micelles (10 mM TC, 2 mM PC) prepared in the absence (A and B, cholesterol free) or the presence of cholesterol (C and D, cholesterol enriched, 0.32 mM). Bile was collected in 15-min intervals, and the flow was determined gravimetrically. Cholesterol content of bile and perfusates was determined by colorimetric assays, and the cumulative rates of cholesterol secretion from the liver (left) and intestine (right) were calculated. The dashed line reflects the rate of cholesterol perfusion (C, D). Data are mean ± SEM and were analyzed by linear regression. Statistical differences in slopes are indicated (n = 8–10). ∗∗*P* < 0.01, ∗∗∗*P* < 0.001, and ∗∗∗∗*P* < 0.0001. PC, phosphatidylcholine; TC, taurocholate.

To determine if there was a compensatory upregulation in intestinal cholesterol secretion associated with the loss of hepatic G5G8 and biliary cholesterol secretion, we employed our previously published method for simultaneous determination of biliary and intestinal cholesterol secretion (15, 24). The rate of biliary cholesterol secretion was reduced in both male and female G5G8^LKO^ mice relative to their littermate controls (Fig. 4A, B). However, we observed no increase in intestinal cholesterol secretion in either sex. We and others who have examined rates of intestinal cholesterol secretion under a variety of conditions have done so using micelles prepared in the absence of cholesterol (cholesterol free). A shortcoming of this approach is that bile is not cholesterol free, even in the absence of hepatic G5G8. Therefore, we prepared mixed micelles that contained cholesterol within the physiological range (cholesterol enriched, Fig. 4 C, D). Again, we observed a substantial reduction in biliary cholesterol secretion in the absence of any change in secretion from the intestine. The rate of cholesterol perfusion is depicted with a dashed line and reflects the theoretical rate of cholesterol appearance in the perfusate after passing through the proximal small intestine had there been no net exchange of cholesterol (Fig. 4C, D). Under the conditions of the present study, we observed a net loss of cholesterol from the perfusate, indicating uptake of cholesterol as the perfusate passed through the proximal small intestine. Nonetheless, genotypic differences were not observed.

**Fig. 5:**
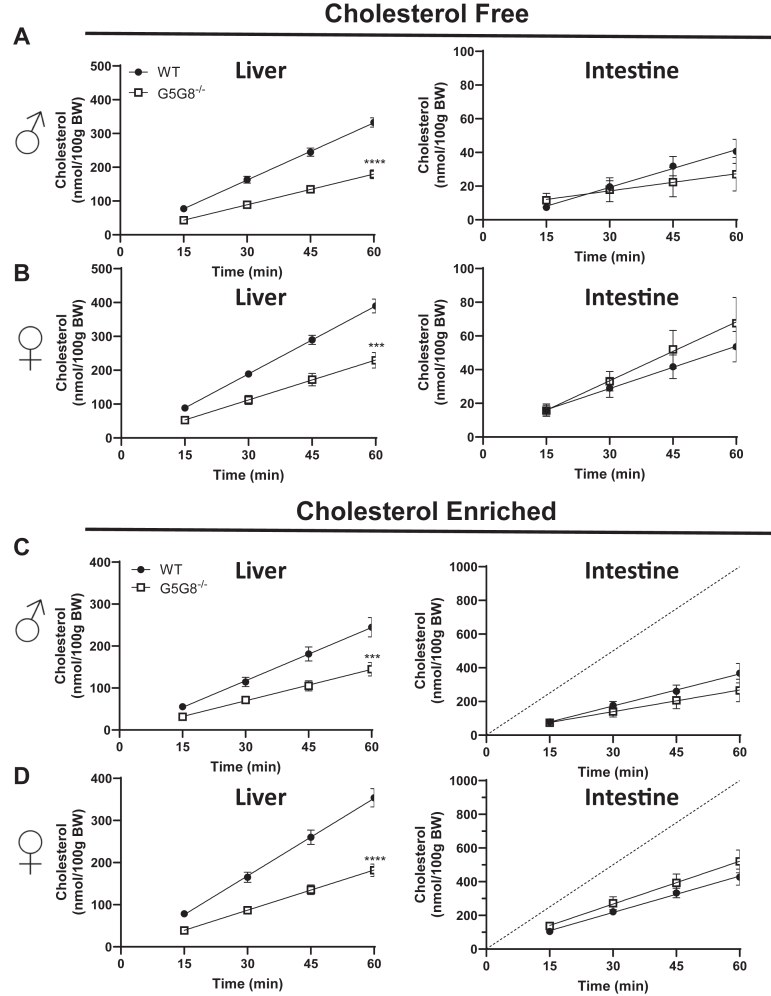
Biliary and intestinal cholesterol secretion in WT and whole-body ABCG5/ABCG8-deficient mice. Mice were maintained on PS-free diets and analyzed at 8–10 weeks of age. The common bile duct was ligated, the gallbladder was cannulated, and the bile was diverted into collection tubes in both male (A, C) and female (B, D) mice. Bile flow was maintained by continuous tail-vein infusion of TC (100 nmol/min). The proximal small intestine (10 cm) was perfused with Krebs-Henseleit buffer containing mixed micelles (10 mM TC, 2 mM PC) prepared in the absence (A and B, cholesterol free) or the presence of cholesterol (C and D, cholesterol enriched, 0.32 mM). The bile was collected in 15-min intervals, and the flow was determined gravimetrically. Cholesterol content of bile and perfusates was determined by colorimetric assay, and the cumulative rates of cholesterol secretion from the liver (left) and intestine (right) were calculated. The dashed line reflects the rate of cholesterol perfusion (C, D). Data are mean ± SEM and were analyzed by linear regression. Statistical differences in slopes are indicated (n = 8–10). ∗∗∗*P* < 0.001, ∗∗∗∗*P* < 0.0001. PC, phosphatidylcholine; TC, taurocholate.

Prior studies of G5G8-independent cholesterol secretion were conducted in mice lacking G5G8 in both liver and intestine. Therefore, we also performed these experiments in whole-body G5G8-deficient mice and their WT littermates (Fig. 5). Our whole-body G5G8-deficient strain is maintained on a PS-free diet in order to prevent confounding effects of biologically active phytosterols present in standard rodent cereal-based diets that vary within and between suppliers as well as to improve breeding efficiency (32, 33). Reductions in biliary cholesterol secretion were observed in G5G8^−/−^ mice in both sexes, regardless of intestinal perfusate. While the regression lines for intestinal cholesterol secretion are not superimposable between genotypes, none of these apparent differences reached statistical significance.

**Fig. 6:**
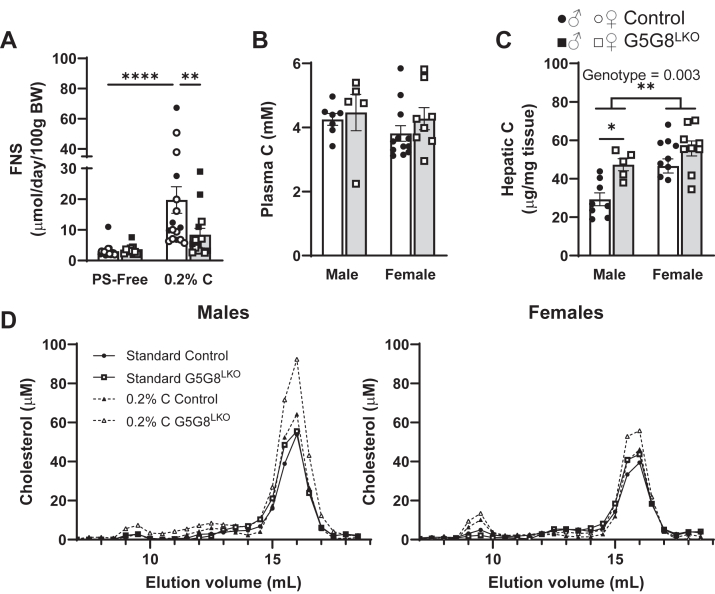
Fecal neutral sterols and plasma and hepatic cholesterol in control and G5G8LKO mice in response to alterations in dietary cholesterol. Mice were bred and housed as in Figure 1. At 10 weeks of age, mice were switched to the PS-free diet for a period of 2 weeks. Mice were then fed the PS-free diet supplemented with cholesterol (0.2% wt/wt) for a period of 2 weeks. Feces were collected over the final 3 days of each diet. Basal bile (30 min) was collected, and tissues were dissected and snap frozen until analysis. A: Fecal neutral sterols in mice were maintained on a control and cholesterol-supplemented diet. Total cholesterol was also determined in terminal plasma (B) and liver (C). D: Plasma was pooled from each group of mice maintained on either the standard diet or the diet supplemented with 0.2% cholesterol, fractionated by FPLC, and cholesterol in each fraction was determined by enzymatic colorimetric assay.

Our data fail to support an adaptive response in the proximal small intestine that elevates the inherent rate of intestinal cholesterol secretion or reduces the rate of cholesterol absorption to compensate for reductions in biliary cholesterol secretion in order to maintain sterol balance. We next asked if TICE could maintain sterol balance in the absence of hepatic G5G8 in the face of a dietary cholesterol challenge. We first placed the mice on the PS-free diet for a period of 2 weeks and measured FNSs (Fig. 6A). As on a standard rodent diet, the absence of hepatic G5G8 had no impact on FNS excretion. Following 2 weeks on the same diet supplemented with cholesterol (0.2% w/w), control mice increased FNS output by 8-fold. G5G8^LKO^ mice appear to partially compensate for the increased dietary cholesterol (3.37 ± 0.48 vs. 8.40 ± 2.11), but the difference between FNS on the PS-free diet compared with the cholesterol-supplemented diet failed to reach statistical significance. Plasma cholesterol was not affected by the loss of hepatic G5G8 on either diet but was elevated in the liver of G5G8^LKO^ mice compared with controls irrespective of sex and statistically significant in males following the dietary cholesterol challenge (Fig. 6B, C). Pooled plasma was then fractionated by FPLC to examine lipoprotein distribution in mice maintained on standard and 0.2% C-containing diets (Fig. 6D). No differences in distribution were observed in either male or female mice maintained on a standard rodent diet. However, we observed a modest increase in the HDL-C in both sexes with the inactivation of hepatic G5G8.

**Fig. 7:**
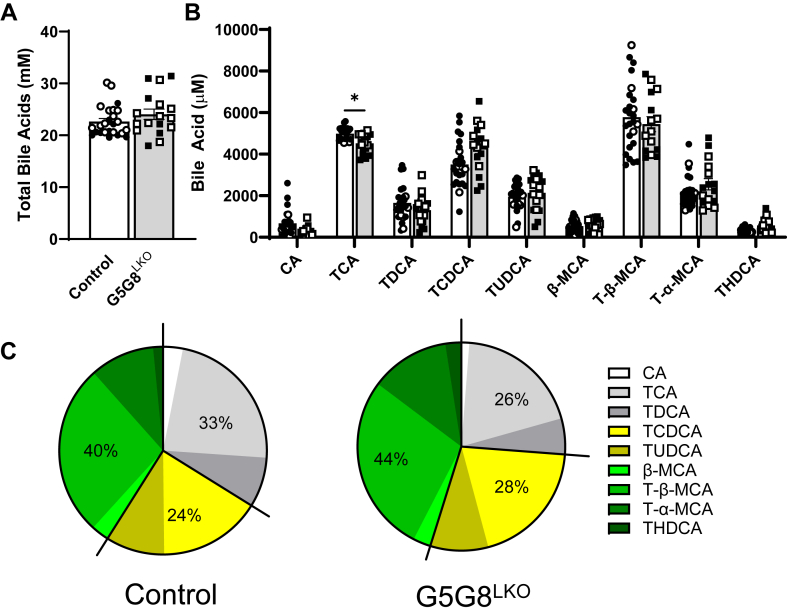
Total bile acids and bile acid profiles in control and G5G8^LKO^ mice in response to alterations in dietary cholesterol. Mice were bred and housed as in Figure 1. At 10 weeks of age, mice were switched to the PS-free diet for a period of 2 weeks. Mice were then fed the PS-free diet supplemented with cholesterol (0.2% wt/wt) for a period of 2 weeks. Basal bile (30 min) was collected and snap frozen until analysis. Bile acids were analyzed by LC-MS/MS. A: All detected bile acids were summed to calculate the concentration of total bile acids. B: Concentrations of individual major bile acids (>1% total) and their metabolites. C: The contribution of the major bile acid classes (cholates, chenodeoxycholates, and muricholates) to the bile acid pool in basal bile. Data are mean ± SEM (n = 6–12) for measures in both male (filled symbols) and female (open symbols) mice and were analyzed by Student's *t*-test (A) or a two-way ANOVA using species and genotype (B) as factors with Sidak’s multiple comparisons tests for effects of genotype. ∗*P* < 0.05.

We next evaluated the bile acid pool in basal bile. Total bile acids were unaffected by hepatic G5G8 deficiency (Fig. 7A). The composition of the bile acid pool was modestly affected by a small reduction in taurocholic acid (Fig. 7B). Total cholates were slightly reduced (33% vs. 26%) in G5G8LKO mice and offset by small increases in both chenodeoxycholates and muricholates (Fig. 7C).

**Fig. 8:**
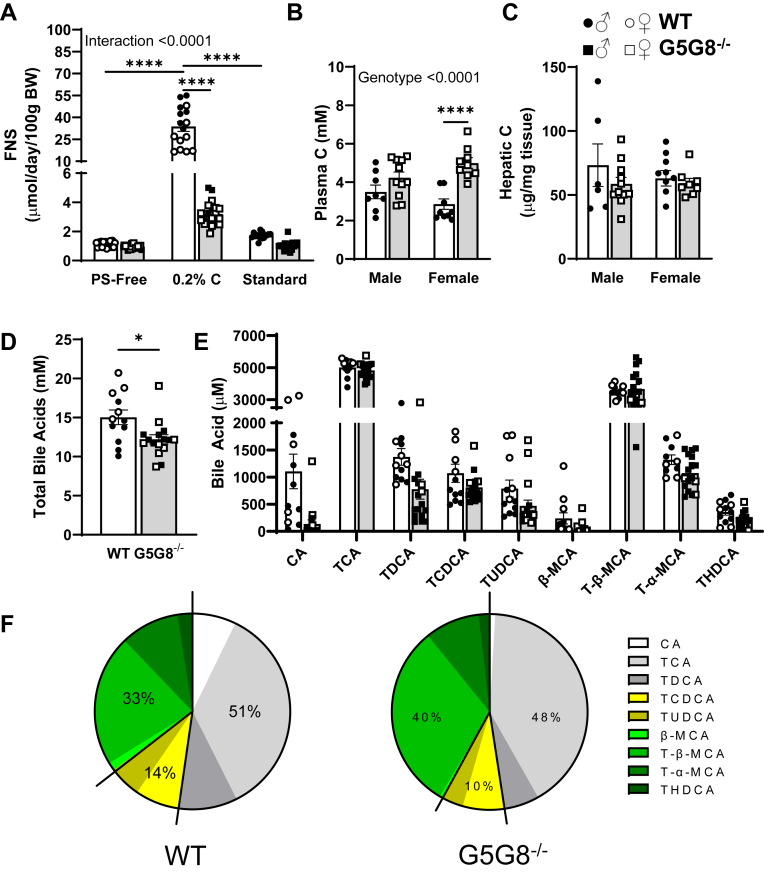
Fecal neutral sterols and bile acid composition of basal bile collected from WT and G5G8^−/−^ mice maintained on multiple diets. Mice were bred and maintained on a PS-free diet up to 10 weeks of age. Baseline feces were collected for 3 days before mice were switched to a PS-free diet supplemented with cholesterol (0.2% wt/wt) for a period of 2 weeks. Mice were then switched to a standard diet for a period of 2 weeks. Feces were collected over the final 3 days of each diet. Basal bile (30 min) was collected, tissues dissected, and snap frozen until analysis. A: Fecal neutral sterols in mice maintained on indicated diet were determined by GC-MS. Total cholesterol was also determined in terminal plasma (B) and liver (C). Bile acids were analyzed by LC-MS/MS. D: All detected bile acids were summed to calculate the concentration of total bile acids. E: Concentrations of individual major bile acids (>1% total) and their metabolites. F: The contribution of the major bile acid classes (cholates, chenodeoxycholates, and muricholates) to the bile acid pool in basal bile. Data are mean ± SEM (n = 6–12) for measures in both male (filled symbols) and female (open symbols) mice and were analyzed by two-way ANOVA using diet and genotype (A), sex and genotype (B and C), or species and genotype (E) as factors with Sidak’s multiple comparisons tests for effects of genotype. *P* values for the effect of genotype irrespective of sex are indicated by text. ∗*P* < 0.05, ∗∗∗∗*P* < 0.0001.

We next analyzed FNS output in WT and G5G8^−/−^ mice at baseline (PS-free diet), following 2 weeks on the cholesterol-supplemented diet, and 2 weeks on a standard rodent diet. The absence of G5G8 had no effect on FNS output on PS-free diet (Fig. 8A). WT mice increased FNS by 33-fold (1.12 ± 0.05 vs. 33.78 ± 3.37, mean ± SEM) following cholesterol feeding, whereas G5G8^−/−^ mice failed to do so (0.96 ± 0.33 vs. 3.2 ± 0.179). Following 2 weeks on a standard diet, genotypic differences in FNS output were no longer observed.

Terminal plasma cholesterol was elevated in G5G8^−/−^ mice compared with WT, regardless of sex, and statistically significant in females despite 2 weeks on a standard rodent diet. Hepatic cholesterol was unaffected by genotype following 2 weeks on standard diet. It should be noted that these terminal measures were conducted with enzymatic colorimetric assays that fail to distinguish cholesterol from phytosterols that likely accumulate in G5G8^−/−^ mice fed a standard diet. The total bile acid pool in basal bile was slightly lower in G5G8^−/−^ mice, but bile acid composition was largely unaffected (Fig. 8D–F). There was a modest increase in total muricholates in G5G8^−/−^ mice at the expense of cholates and chenodeoxycholates.

**Fig. 9:**
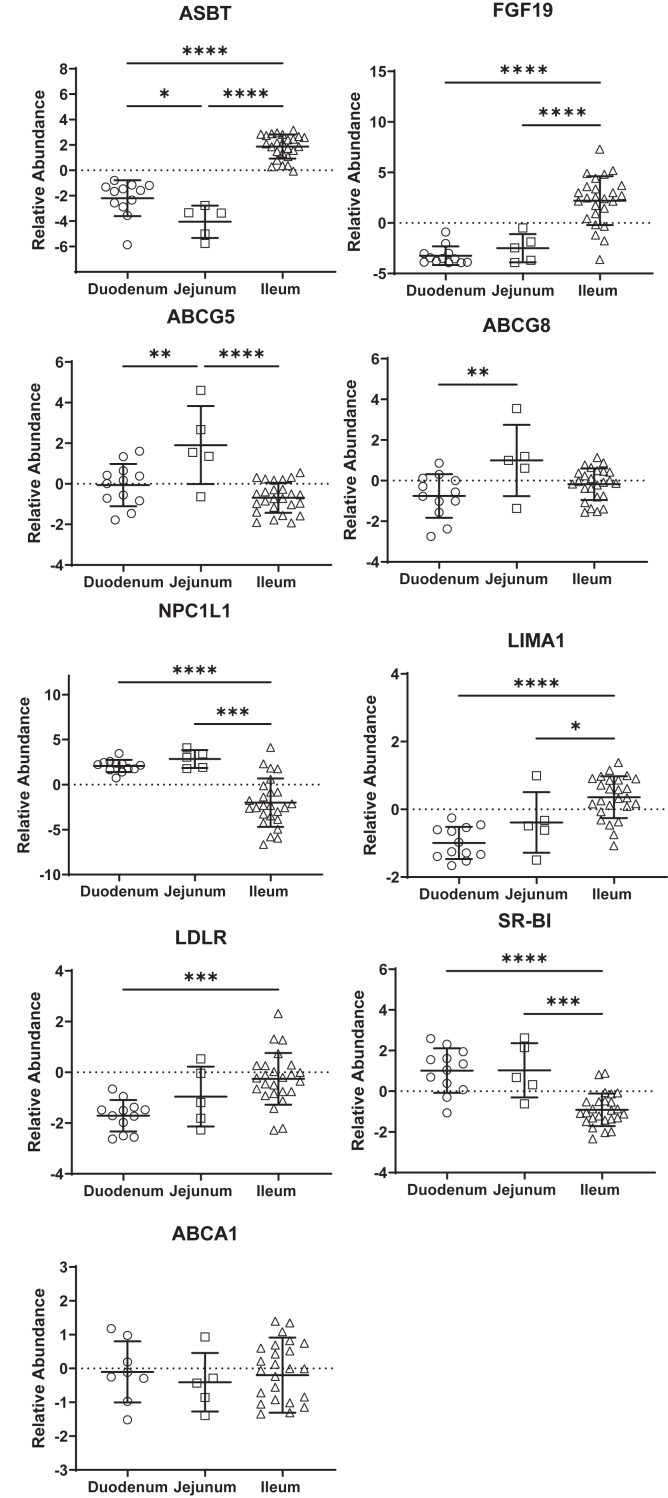
The relative abundance of transcripts encoding sterol-transporting enzymes along the length of the human small intestine. Intestinal tissue was collected from the margins of resected bowel deemed healthy by the surgeon from patients undergoing bowel resection. The relative abundance of transcripts was analyzed by RT-PCR and normalized to the mean Ct values for GAPDH (ΔCt). The relative abundance across segments was normalized to the geometric mean of all ΔCt values for a given transcript, log2 transformed, and expressed as relative abundance. Data were analyzed by one-way ANOVA and post hoc Tukey’s multiple comparisons test to determine differences in transcript abundance between segments. ∗*P* < 0.05, ∗∗*P* < 0.01, ∗∗∗*P* < 0.001, and ∗∗∗∗*P* < 0.0001.

Stable isotope studies have demonstrated that TICE is an active pathway in humans and is thought to mediate ezetimibe-dependent increases in fecal sterol loss (19). TICE is most active in the proximal small intestine of the mouse, but little is known about the mechanisms that mediate intestinal cholesterol secretion in humans and if they vary by intestinal segment. To develop a further understanding of the mechanisms that might mediate TICE in humans, we obtained intestinal tissue from patients undergoing small bowel resections (Table 2). Tissue deemed “healthy” by the surgeon was trimmed from the ends of the resected segment, and the relative abundance of transcripts encoding established sterol-transporting proteins was analyzed by RT-PCR (Fig. 9). We first analyzed the expression of the sodium/bile acid cotransporter (ASBT, SLC10A2) and fibroblast growth factor 19, as both are known to be abundant in the terminal ileum, where they function to facilitate bile acid reabsorption and regulate hepatic bile acid synthesis, respectively. Conversely, NPC1L1 was lower in the distal small intestine relative to the duodenum and jejunum. In mice, LIMA1 is reported to serve as an escort protein for NPC1L1 and facilitate cholesterol absorption (34). However, its expression pattern is opposite that of NPC1L1 and more abundant in the ileum relative to the proximal segments. ABCG5 and ABCG8 were generally most abundant in the jejunum, both transcripts being more abundant than in the duodenum. ABCA1 did not display regional differences in expression along the small intestine. Whereas the LDL receptor (LDLR) was modestly elevated in the ileum relative to other segments, the HDL receptor, scavenger receptor class B type I, was lower in the ileum.

## Discussion

The present series of experiments is consistent with the prior literature demonstrating that a reduction in biliary cholesterol secretion has no effect on cholesterol balance in mice maintained on standard diets. However, our results do not support a compensatory upregulation of TICE as an adaptive response to compromised biliary cholesterol secretion. Intestinal cholesterol secretion rates were unaffected by the loss of G5G8 in the liver or in both the liver and intestine. Further, we observed no reduction in intestinal cholesterol content along the length of the small intestine in mice lacking hepatic G5G8. van de Peppel *et al*. (35) demonstrated bidirectional flux of cholesterol in which a substantial portion of cholesterol secreted by the intestine is efficiently reabsorbed, indicating competing absorptive/reabsorptive and secretory processes across the intestinal epithelium. We conclude that the arrival of cholesterol-poor bile in the proximal small intestine favors the loss of cholesterol from intestinal enterocytes and closes the gap in intestinal sterol content due to the reduction in biliary cholesterol secretion.

The constant rate of intestinal cholesterol secretion was observed between genotypes regardless of the presence of cholesterol in mixed micelles in the intestinal perfusate. It should be noted that in the experiment using cholesterol-enriched perfusates, less cholesterol exited the first 10 cm of the small bowel than entered. Thus, a fraction of cholesterol was taken up by the intestinal epithelium, indicating net absorption. Importantly, the rate of apparent absorption did not differ between genotypes in this acute setting. These results are consistent with our gene expression data, which show no difference in NPC1L1 mRNA. Ezetimibe, which inhibits NPC1L1 and reduces cholesterol absorption by greater than 70% in rodents, is also a robust stimulus for TICE in a G5G8-dependent manner (19, 36, 37, 38).

Comparing the magnitude of the difference between the G5G8^LKO^ and G5G8^−/−^ mice to their respective littermate controls, intestinal G5G8 partially compensates for the loss of hepatic G5G8 in mice challenged with dietary cholesterol but is incapable of maintaining sterol output under these experimental conditions. This may be due, in part, by the expected alterations in intestinal gene expression following cholesterol feeding. Dietary cholesterol represses Ldlr and upregulates the inducible degrader of the LDLR (IDOL) and Abca1 expression via liver X receptor (Lxr, Nr1h3/Nr1h2 (39)). The combined effect of these changes favors less intestinal uptake of cholesterol from the plasma compartment and promotes the formation of intestinally derived HDLs. Indeed, we observe an increase in plasma HDL following cholesterol feeding that is exacerbated by the absence of hepatic G5G8.

In mice maintained on a standard diet, we observed a modest increase in the expression of Ldlr but not other Srebp2 target genes, such as Hmgcr or Hmgcs, in the intestine of G5G8^LKO^ mice. This could theoretically increase uptake of cholesterol from apoB-containing lipoproteins from the plasma compartment and facilitate transcellular transport of cholesterol across the enterocyte. However, acute reductions in Ldlr decrease TICE, whereas Ldlr-deficient mice displayed increased TICE relative to WTs (16). Regardless, the secretion of cholesterol by the proximal small intestine was unaffected by the absence of hepatic G5G8.

In the liver, we saw genotypic effects for a reduction in Hmgcr and Hmgcs but not Ldlr. These data suggest a modest repression of the cholesterol biosynthetic pathway, presumably mediated by reductions in Srebp2 processing. This was not the case for Cyp7a1 or Srebp1c, suggesting sterol accumulation in hepatocytes in the absence of G5G8 did not stimulate the Lxr signaling. We observed sex differences in the abundance of transcripts associated with cholesterol and bile acid synthesis (Fig. 3). Independent of genotype, transcripts encoding the cholesterol biosynthetic machinery were generally increased in females compared with males. The abundance of Cpy7a1 was generally higher in females, whereas Cyp8b1 was reduced. However, sex effects were not detected in hepatic cholesterol, total bile acids, or individual bile acid species analyzed by LC-MS/MS (Figs. 7 and 8). Thus, the biological significance of these observations is unclear.

In our time-course study of acute liver G5G8 deficiency, we do not know how rapidly genetic inactivation of *Abcg5 Abcg8* resulted in a loss of G5G8 protein and biliary cholesterol secretion. Our first measure of FNS excretion was 3 days following AAV delivery. We observed no difference in FNS at any point up to 28 days. Biliary cholesterol secretion was clearly reduced in bile collected at termination of the experiment. Thus, the adaptation to hepatic G5G8 deficiency is seamless with no apparent period of reduced cholesterol elimination followed by recovery. We also observed no differences in total fecal bile acids or the concentration of bile acids in basal bile. While differences in bile acid composition in both liver-specific and whole-body knockout mice favor less hydrophobic bile, it is unclear if this difference would be sufficient to impact TICE. In prior perfusion studies of TICE, hydrophilic and hydrophobic bile acids had similar effects on the rates of TICE, whereas phospholipid content in the perfusate had a more substantial effect (12). The composition of our model bile did not examine other bile acids, which may have some effect on apparent rates of cholesterol secretion.

To this point, it has been assumed that TICE can compensate for disruptions in biliary cholesterol secretion. Indeed, we observe this in mice maintained on a standard diet and a purified diet containing trace amounts of cholesterol. However, the presence of cholesterol at 0.2% in the diet overwhelmed TICE or other adaptive responses to maintain sterol output and prevent accumulation in plasma and tissues. While TICE is both measurable and can be stimulated pharmacologically, it is incapable of providing a defense against dietary cholesterol in the absence of G5G8. This may or may not be true in humans. Hypercholesterolemia is not always observed in the clinical presentation of *STSL*. This may simply reflect the amount of cholesterol in the diets of *STSL* patients. However, it may be the case that TICE offers some protection from hypercholesterolemia in sitosterolemics, provided their diet is sufficiently low in animal fat and does not overwhelm this mechanism.

Stimulating TICE is an attractive target for accelerating RCT and reducing the risk of atherosclerotic cardiovascular disease in patients with established plaque burden. TICE bypasses the hepatobiliary pathway and would not be expected to increase the risk of gallbladder disease. Given the prominent role of G5G8 in biliary cholesterol secretion, it is not surprising that variants in *ABCG5* and *ABCG8* have also been linked to cholesterol gallstones. While TICE can be stimulated by pharmacological agents, this has not been demonstrated in the absence of G5G8. Genetic and pharmacological approaches for intestinal-specific stimulation of the LXR pathway have been shown to accelerate RCT and reduce atherosclerosis in mouse models. Thus, the stimulation of G5G8-dependent TICE offers a viable path forward to accelerate cholesterol excretion in the absence of increased gallstone risk.

TICE is reported to be most active in the proximal small intestine in mice (12). Consistent with this, our data demonstrate that sterol content in the intestinal wall and lumen do not differ during the digestive phase in the first ∼6 cm, despite the reduction in biliary cholesterol secretion. Our survey of intestinal expression of sterol-transporting enzymes in human tissue suggests that the pathway may be more prominent in the jejunum. Expression of ABCG5 and ABCG8 was most abundant in this segment of the small bowel, whereas expression of LDLR was lowest in the duodenum. However, the relative expression of scavenger receptor class B type I was higher in the proximal bowel relative to the ileum, and both LDL and HDL were shown to be donors to TICE in human jejunum in Ussing chamber preparations (16). LIMA1 was previously reported to serve as an escort protein for NPC1L1 in mice, and a frameshift variant in the gene was identified in Chinese kindred with low LDL-C and cholesterol absorption (34). However, transcripts encoded by these genes demonstrated an opposite expression pattern in the human intestine. The interpretation of these data is limited by the fact that we are only reporting levels of mRNA and that NPC1L1 activity is also post-translationally regulated by intracellular trafficking between the cell surface and an intracellular pool in a sterol-dependent manner.

## Data availability

All data will be retained and made available upon request in accordance with institutional policies at the University of Kentucky.

## Supplemental data

This article contains supplemental data.

## Uncited figure

Figure 9.

## Conflict of interest

The authors declare that they have no conflicts of interest with the contents of this article.
