# Supplementary material for: ABCG5/ABCG8-independent mechanisms fail to maintain sterol balance in mice fed a high-cholesterol diet
Source: J Lipid Res. 2025 Sep 16;66(10):100902. doi: 10.1016/j.jlr.2025.100902 (PMC12550799; doi:10.1016/j.jlr.2025.100902)
Supplement: Supplemental Material [file mmc1.docx]

**Supplemental Material**

**Table S1.** Primer Sequences for rtPCR analysis of transcript abundance

| Target | Sequence |
| --- | --- |
| Srebp1c | F: 5’ – GGA GCC ATG GAT TGC ACA TT – 3’  R: 5’ – GGC CCG GGA AGT CAC TGT – 3’ |
| Fas | F: 5’ – GCT GCG GAA ACT TCA GGA AAT – 3’  R: 5’ – AGA GAC GTG TCA CTC CTG GAC TT – 3’ |
| Gapdh | F: 5’ – TGT GTC CGT GGA TCT GA – 3’  R: 5’ – CCT GCT TCA CCA CCT TCT TGA T – 3’ |
| Acc1 | F: 5’ – TGG ACA GAC TGA TCG CAG AGA AAG – 3’  R: 5’ – TGG AGA GCC CCA CAC ACA – 3’ |
| Scarb1 | F: 5’ – GAT GGG ACA TGG GAC ACG AAG CCA – 3’  R: 5’ – TCT GTC TCC GTC TCC TTC AGG TCC TGA – 3’ |
| Abcg5 | F: 5’ – CCT TTA AAG CAA CCG TGT CG – 3’  R: 5’ CTT AGA CAG AGC CAA AGC CT – 3’ |
| Abcg8 | F: 5’ – CTG TGG AAT GGG ACT GTA CTT – 3’  R: 5’ – CTA TGA GAC CTC CAG GGT ATC T – 3’ |
| Hmgcs | F: 5’ – GAG CTG GGC AGA CAT GTT AT – 3’  R: 5’ – GAA GAG ACA TAG GCA GGG TTT AG – 3’ |
| Hmgcr | F: 5’ – CTT GTG GAA TGC CTT GTG ATT G – 3’  R: 5’ – AGC CGA AGC AGC ACA TGA T – 3’ |
| Ldlr | F: 5’ – AGG CTG TGG GCT CCA TAG G – 3’  R: 5’ – TGC GGT COA GGG TCA TCT – 3’ |
| Npc1l1 | F: 5’ – TGG ACT GGA AGG ACC ATT TCC – 3’  R: 5’ – GCG CCC CGT AGT CAG CTA T – 3’ |
| Abca1 | F: 5’ – CGT TTC CGG GAA GTG TCC TA – 3’  R: 5’ – GCT AGA GAT GAC AAG GAG GAT GGA – 3’ |
| Cyp7a1 | F: 5’ – GCT GTC TGG GTC ACG GAA GG – 3’  R: 5’ – AAG TGA ATA GGG ACG CCC GC – 3’ |
| Cyp8b1 | F: 5’ – GCC TTC AAG TAT GAT CGG TTC CT – 3’  R: 5’ – GAT CTT CTT GCC CGA CTT GTA GA – 3’ |
